# Updated Review of the Toxicity of Selected *Fusarium* Toxins and Their Modified Forms

**DOI:** 10.3390/toxins13110768

**Published:** 2021-10-29

**Authors:** Adam Pierzgalski, Marcin Bryła, Joanna Kanabus, Marta Modrzewska, Grażyna Podolska

**Affiliations:** 1 Department of Food Safety and Chemical Analysis, Prof. Waclaw Dabrowski Institute of Agricultural and Food Biotechnology-State Research Institute, Rakowiecka 36, 02-532 Warsaw, Poland; marcin.bryla@ibprs.pl (M.B.); joanna.kanabus@ibprs.pl (J.K.); marta.modrzewska@ibprs.pl (M.M.); 2 Department of Cereal Crop Production, Institute of Soil Science and Plant Cultivation–State Research Institute, Czartoryskich 8, 24-100 Puławy, Poland; aga@iung.pulawy.pl

**Keywords:** toxicity, *Fusarium* toxins, modified mycotoxins metabolism, deoxynivalenol, T-2 toxin, zearalenone

## Abstract

Mycotoxins are one of the most dangerous food and feed contaminants, hence they have significant influence on human and animal health. This study reviews the information reported over the last few years on the toxic effects of the most relevant and studied *Fusarium* toxins and their modified forms. Deoxynivalenol (DON) and its metabolites can induce intracellular oxidative stress, resulting in DNA damage. Recent studies have also revealed the capability of DON and its metabolites to disturb the cell cycle and alter amino acid expression. Several studies have attempted to explore the mechanism of action of T-2 and HT-2 toxins in anorexia induction. Among other findings, two neurotransmitters associated with this process have been identified, namely substance P and serotonin (5-hydroxytryptamine). For zearalenone (ZEN) and its metabolites, the literature points out that, in addition to their generally acknowledged estrogenic and oxidative potentials, they can also modify DNA by altering methylation patterns and histone acetylation. The ability of the compounds to induce alterations in the expression of major metabolic genes suggests that these compounds can contribute to the development of numerous metabolic diseases, including type 2 diabetes.

## 1. Introduction

Mycotoxins are secondary metabolites biosynthesized by fungi and can contribute to the induction of toxic effects in humans and animals [1]. The most important mycotoxins of the *Fusarium* genus are deoxynivalenol (DON), T-2 toxin, and zearalenone (ZEN). These compounds are produced during fungal infection of plants, and they weaken and colonise the host plants. This process is useful, as living plant tissues show protective mechanisms that impede parasitic fungal growth. Other *Fusarium* phytotoxins include fumonisins, fusarins, enniatins, and beauvericinis [2,3]. *Fusarium* fungi infect several plants, including crops, such as maize, wheat, oats, rye, barley, and pastures [4,5,6]. In EU countries, the maximum content of *Fusarium* mycotoxins in unprocessed cereals is specified in the regulation 1881/2006 [7]. For maize, these standards have been updated in Regulation 1126/2007 [8]. For T-2 and HT-2 toxins, the maximum levels in unprocessed cereals were described in Recommendation 2013/165/EU [9]. These standards are presented in Table 1.

The effects of global warming, such as increasing temperature and rainfall levels, play a vital role in the infestation of pathogenic fungi. Several studies have demonstrated that these effects induce enhanced colonisation and toxinogenic potency of fungi from the *Fusarium* genus [10,11,12,13,14,15].

In food materials, such as cereals, the ambient conditions during food processing do not enable complete degradation of mycotoxins. These toxins can therefore infest food in great quantities, which is a direct risk to human health [16,17].

**Figure 1:**
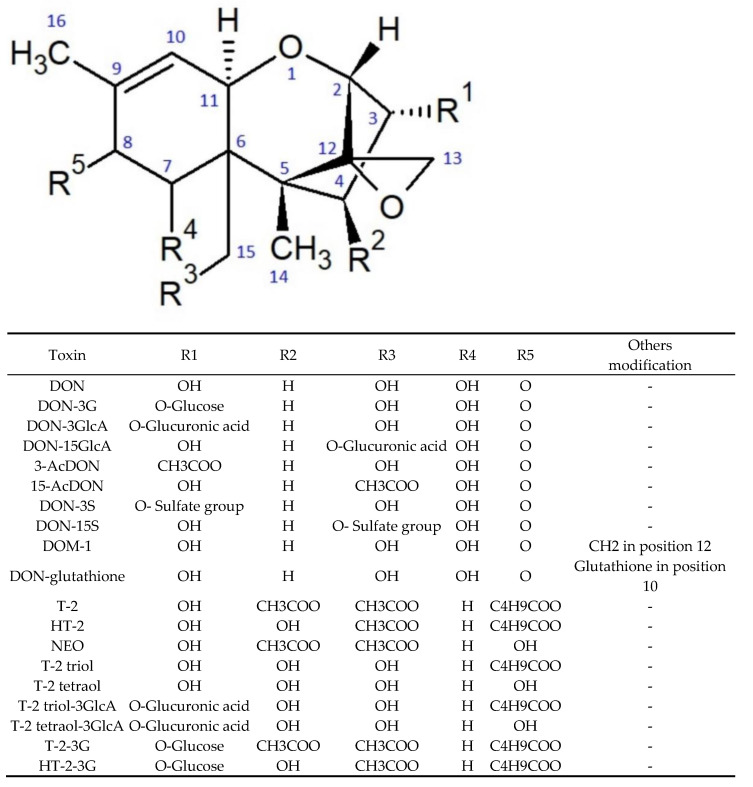
Chemical structure of trichothecenes its modified forms.

**Figure 2:**
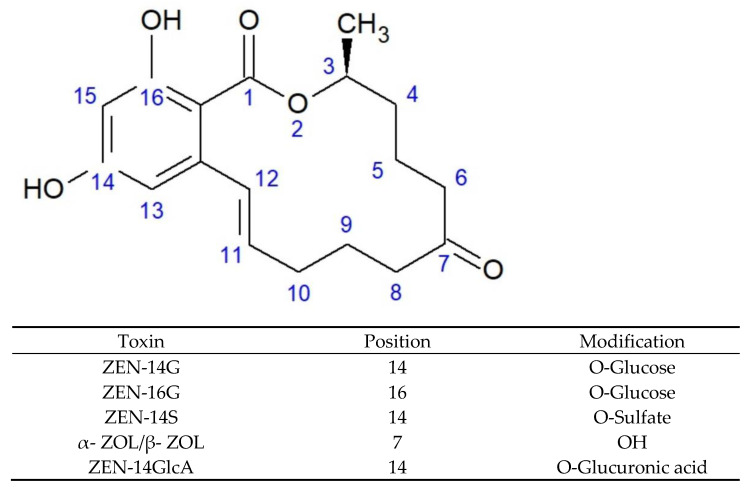
Structure of zearalenone and its modified forms.

DON and T-2 toxin are trichothecenes, which are all characterised by the presence of a triple ring 12,13-epoxytrichotec-9-ene skeleton. Trichothecenes comprise three groups, differentiated according to a substituent linked at the C-8 position, with a separate group (D) consisting of compounds that contain a macrocyclic linkage between the C-4 and C-15 atoms. Group A trichothecenes can have both methylene and hydroxyl groups at the C-8 position or, as in the case of T-2 toxin, an ester group. Group B includes compounds that have a keto group at this position (DON, nivalenol, and fusarenone X). Moreover, group C trichothecenes are characterised by the presence of epoxide at the C-7 or C-8 position [18,19]. The chemical structures of the selected trichothecenes are shown in Figure 1. In contrast to trichothecenes, ZEN is a macrocyclic β-resorcyclic lactone. This toxin has a heterocyclic structure composed of two hydroxyl and carbonyl groups and a methyl group [20]. The chemical structures of ZEN and its main metabolites are shown in Figure 2.

**Figure 3:**
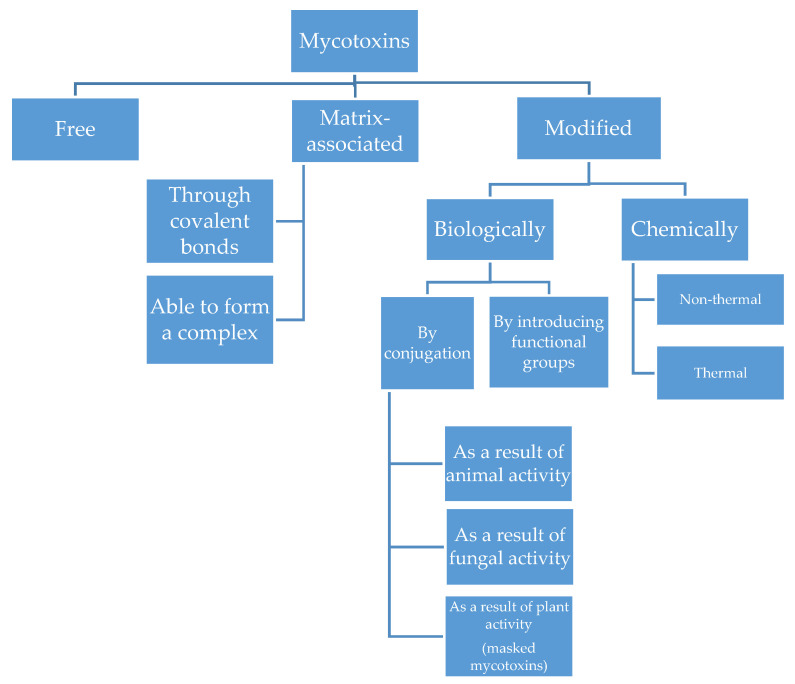
Taxonomy of Mycotoxins [21].

Mycotoxins can undergo modifications due to their environment and activity. The literature focuses mainly on mycotoxin metabolites produced during detoxification reactions in plants. Frequently referred to as ‘masked mycotoxins’, the compounds of this group remain undetected during routine food tests conducted to determine the mycotoxin content. The term ‘masked mycotoxins’ applies only to the plant metabolites of mycotoxins, and it does not include compounds formed as a result of the metabolic activities of bacteria, fungi, and animals. Multiple studies conducted in recent years have led to the discovery of a substantial number of previously unknown derivatives of mycotoxins, and different approaches have been adopted to classify these compounds. At present, the most commonly accepted taxonomy is that developed by M. Rychlik et al. in 2014, as shown in Figure 3 [21,22,23]. Masked mycotoxins formed in the course of the metabolism of the parent compound are often characterised by reduced or no toxicity. Depending on the mycotoxin, this process can include various mechanisms, such as loss of functional groups, which can reduce toxicity and bioavailability [24]. Nevertheless, existing research indicates that mycotoxin derivatives could have higher toxicity than their basic analogues. One of the principal issues in assessing the toxicity of mycotoxins in food is the highly probable in vivo interactions that can occur between parent toxins and their metabolites. They are likely to increase the toxicity of these compounds by inducing synergistic effects. Some mycotoxin derivatives are also absorbed in the intestines to a much greater extent than the parent mycotoxins [25,26,27].

The toxicity of DON, T-2 toxin, and ZEN has been well explored and discussed in numerous publications. However, reports on the toxicity of the modified forms of these compounds are limited. Moreover, unknown metabolites of *Fusarium* toxins are still being discovered. The investigation of the properties of these compounds is required as parent toxins can be modified chemically both in vivo and in vitro and exert an influence on cells [23,28,29]. The majority of published studies on the toxicity of modified *Fusarium* mycotoxins are based on cell exposure to the tested compounds and the use of cytotoxicity tests, such as MTT or neutral red assays. The inhibitory concentration value, IC_50_, indicates the concentration of the tested toxin at which cell proliferation decreases by 50% [30,31,32,33,34,35,36]. Another common method of toxicity assessment use in vivo models (usually porcine) to observe the toxic effects induced by toxins [37,38]. However, these aforementioned methods have significant limitations, as it is not possible to compare the mechanisms associated with toxic effects of parent toxins and those associated with their modified forms. Over the last few years, several studies, which involved molecular biology techniques and in silico analyses, have been aimed at gaining insight into some aspects of toxicity shown by modified *Fusarium* toxins [32,36,39,40,41,42,43,44,45,46,47]. Numerous studies, which assessed the cytotoxicity of these compounds by using different cell lines and approaches and evaluated the influence of the interaction between toxins on the intensity of their induced effects, have also been published [32,33,35,39,48]. This review aims to summarise and compare the results of recent toxicity studies on modified *Fusarium* toxins and their parent forms.

## 2. Metabolism

### 2.1. DON

Many DON metabolites are specific to their respective fungi, plants, and animals. Some DON biotransformation routes are identical in various organisms; hence, they produce the same metabolites. Such metabolites include DON-3S, which is produced by both plants and poultry, and DON-3G, the plant metabolite of DON, which has recently been confirmed to be produced by aphids [49,50,51]. *Fusarium* fungi are capable of DON acetylation, resulting in the formation of 3-acetyl-deoxynivalenol (3-AcDON) or 15-acetyl-deoxynivalenol (15-AcDON) [52]. The type of fungi-generated acetylated derivatives is determined genetically, and the fungi are differentiated into chemotypes of 3-AcDON or 15-AcDON [53].

The most common DON metabolite in plants is deoxynivalenol-3-glucoside (DON-3G). It is a product of detoxification in plants exposed to DON. In comparison with its parent toxin, DON-3G has increased polarity due to the introduction of a glucose molecule, and it is stored in vacuoles in this form [22,54]. Furthermore, the formation of DON-GSH from the conjugation of DON with glutathione has been recently described. This compound undergoes further degradation into DON-S-cysteine (DON-S-Cys), DON-S-cysteinyloglicyne (DON-S-Cys-Gly), and DON-2H-GSH [55]. Plant metabolites of DON-containing sulfates have also been reported, including deoxynivalenol-3-sulphate (DON-3S) and deoxynivalenol-15-sulphate (DON-15S), which were identified in wheat ears previously inoculated with *Fusarium graminearum* [56]. Transgenic wheat varieties characterised by increased resistance to *Fusarium* infections can also contain 3-AcDON [57]. These metabolites are those most often found in the literature, but the entire DON biotransformation process produces a significantly larger number of metabolites that are only occasionally reported.

DON in humans and animals is mostly excreted as glucuronides. Glucuronidation reactions mainly take place in the liver, in the presence of uridine-glucuronosyltransferases (UGT), leading to the formation of DON-3-*O-*glucuronide (DON-3GlcA) and DON-15-*O*-glucuronide (DON-15GlcA). In humans, the main product of glucuronidation, which occurs in the liver, is DON-15GlcA. Moreover, coupling with glucuronide can occur at a lower efficacy in the intestines and kidney [58,59,60]. In vivo DON can also be metabolised to de-epoxy-deoxynivalenol (DOM-1) in a process that involves the participation of faecal bacteria. This transformation is not observed in all humans, as it is highly dependent on the composition of the intestinal bacterial flora [38,61]. Similar to DON, this compound undergoes glucuronidation to form deepoxy-deoxynivalenol-15-glucuronide (DOM-15-glucuronide) and deepoxy-deoxynivalenol-3-glucuronide (DOM3-glucuronide) [60]. Recent studies carried out on broiler chickens proved that they are able to metabolise DON to DON-3S [49]. It has also been reported that this compound is the main DON metabolite in the eggs of laying hens [50]. Both DON-3S and DON-15S were also identified in the liver cells of rats that had been previously administered DON intraperitoneally [62]. It has been recently reported that aphids are able to metabolise DON into DON-3G [51]. However, this pathway of DON biotransformation has not been described in other animals.

### 2.2. T-2 and HT-2 Toxins

The most common metabolite of the T-2 toxin is the HT-2 toxin, which is formed through the deacetylation of the parent toxin. HT-2 is a fungal metabolite of the *Fusarium* genus, but it can also be produced as a result of metabolism in plants and humans. *Fusarium* can also metabolise T-2 and HT-2 to T-2-3-glucoside (T-2-3G) and HT-2-3-glucoside (HT-2-3G), respectively [63]. The ability to transform T-2 to T-2-3G has also been demonstrated for some yeast species of the Blastobotrys genus. The Blastobotrys genus is also characterised by its ability to metabolise T-2 to 3-acetyl T-2 and neosolaniol (NEO) [64].

T-2-3G and HT-2-3G are also formed through the biotransformation of T-2 and HT-2 in plants. In plants, this transformation is the main detoxification pathway for these toxins. Furthermore, plants may be able to metabolise T-2 and HT-2 to their respective di-, tri-, and tetraglucosides [65]. Some plants may be able to transform the T-2 toxin into HT-2 toxin, T-2 tetraol, 3′-hydroxy-T-2, and 3′-hydroxy-HT-2. This ability was observed in the shrub genus Baccharis, which is known for its relatively high resistance to group A trichothecenes [66]. New plant metabolites of T-2 and HT-2 have been identified in recent studies involving wheat-derived root and leaf cell cultures. The identified compounds included glucosides of T-2 triols and tetraols, acetyl and hydroxylated metabolites, hexose-pentose conjugates, and malonyloglucosides [67].

In humans and animals, T-2 toxin (T-2) is metabolised rapidly and efficiently to HT-2 toxin (HT-2) through hydroxylation [68]. Hydroxylation takes place in the intestines, liver, and plasma, as well as in other organs and tissues. Moreover, T-2 can be transformed into HT-2 as a result of intestinal microflora activity [69,70]. The metabolism of T-2 in animals varies considerably. In rats, T-2 can be hydroxylated to HT-2 or NEO, and these compounds are transformed to T-2 tetraol (HT-2 indirectly through 15-acetyl-tetraol). The identified T-2 metabolites in rodents include derivatives containing a hydroxyl group (3′-hydroxy-T-2,3′-hydroxy HT-2), derivatives without an epoxy group (deepoxy-3′-hydroxy-HT-2, deepoxy-3′-hydroxy-T-2 triol, deepoxy-T-2 tetraol, and deepoxy-15-acetyl-T-2 tetraol), as well as glucuronide conjugates (HT-2, T-2 tetraol, and 3′-hydroxy-HT-2). Similarly, in cattle, the metabolites include 3′-hydroxy-T-2, 3′-hydroxy-HT-2, and deepoxy-T-2, and substantial quantities of acetyl-T-2, acetyl-HT-2, and 3-acetyl-3′-hydroxy-HT-2. In pigs, the main T-2 metabolites include 3′-hydroxy-HT-2, T-2 triol, and numerous glucuronides: T-2, 3-hydroxy-T-2, NEO, 4-deacetylneosolaniol HT-2, 3-hydroxy HT-2, T-2 triol, and T-2 tetraol. 3′-hydroxy-HT-2 is also the main T-2 metabolite in chickens. In animals, T-2 triol, T-2 tetraol, 4-acetoxy-T-2 tetraol, 8-acetoxy-T-2 tetraol, and 15-acetoxy-T-2 tetraol were identified [69]. Based on animal and in vitro tests, T-2 metabolites in humans include NEO, T-2 triol, T-2 tetraol, 3′-hydroxy-T-2, and 3′-hydroxy-HT-2 [69,71].

### 2.3. ZEN

A considerable number of ZEN metabolites are produced by organisms from different kingdoms; for example, α-zearalenol (α-ZOL) and β-zearalenol (β-ZOL) can be produced by fungi, plants, and animals. Animals and fungi can reduce these compounds to α-zearalanol (α-ZAL) and β-zearalanol (β-ZAL). Zearalenone sulphate (ZEN-14S) and zearalenone-14-O-β-glucoside (ZEN-14G) are common to both plants and fungi [72,73,74,75,76,77]. Fusarium fungi can metabolise ZEN into ZEN-14S, and this ability has also been observed for the fungal genera Rhizopus, Aspergillus, and Trichoderma [72,73]. Other ZEN metabolites produced by Fusarium are α-ZOL, β-ZOL, α-ZAL, β-ZAL, and zearalanone (ZAN), the presence of which has been reported in maize stems and in a *Fusarium* culture kept on rice substrate [74,78]. ZEN has been recently observed to be efficiently biotransformed by fungi of the Trichoderma genus, which transforms this compound, among others, to ZEN-14G [73]. Yeasts are also able to metabolise ZEN; it has been proven that several strains of the genera Pichia, Brettanomyces, Hansenula, Schizosaccharomyces, Candida, and Saccharomycopsis can reduce ZEN to α-ZOL [79].

The best-known ZEN metabolites in plants are ZEN-14G, also referred to as zearalenone-4-O-β-glucoside, and α-ZOL and β-ZOL with their glucosides [75,80]. The results of the studies carried out on suspended cultures of wheat cells showed that the reaction products of plant glycosylation can also include ZEN-16-glucoside (ZEN-16G) and ZEN malonylo-glucosides [81]. Plants may be able to metabolise ZEN to ZEN-14S, as seen in *A. thaliana*, but no studies have reported this observation in cereals [80].

In humans and animals, Phase I ZEN metabolites include α-ZOL and β-ZOL, which are formed through ZEN hydroxylation. This is followed by the coupling reactions of ZEN and its reduced forms, resulting in the formation of their respective glucuronides and sulfates. α-ZOL and β-ZOL can undergo further reduction to form α-ZAL and β-ZAL, which also undergo glucuronidation. α-ZAL is also known to be transformed into β-ZAL or ZAN in vivo. An analysis of human urine samples indicated that the main ZEN metabolites were ZEN-GlcA and α-ZAL-GlcA [76,77,79].

## 3. Modified Forms of DON

### 3.1. In Vitro Cytotoxicity

The available data on the assessment of DON and its modified forms for cytotoxicity are not clear. Depending on the methods applied, cell lines, toxin concentrations, and exposure times, the results obtained varied [25,30,31,39,41,43,82]. Some studies have pointed to higher cytotoxicity of DON compared with that of its acetylated forms. The study by A. Juan-García et al. reported that the IC_50_ value for DON in HepG2 liver cell line was 4.3 (μmol/L), whereas those for 3-AcDON and 15-AcDON were 6.2 and 8.1 (μmol/L), respectively [30]. Similarly, a study on GES-1 human stomach cell line reported relatively high DON toxicity, with the following ranking of cytotoxicity proposed: DON > 15-AcDON >> 3-AcDON > DON-3G. It is worthy of note that the cells exposed to 3-AcDON had high longevity, which was only slightly lower than that observed in cells exposed to DON-3G, which implies that these compounds are not toxic to stomach cells [31]. Further studies conducted by the same author on the same cell line also showed that DON had higher toxicity than 15-AcDON [41]. However, data supporting contradictory conclusions have been reported. Moreover, of all the acetylated DON metabolites, the most toxic compound is still unknown. For instance, studies that used the HepG-2 cell line reported that 3-AcDON had higher cytotoxicity than DON and 15-AcDON, whereas studies on the Caco-2 cell line (small intestinal cells) reported that 3-AcDON showed the lowest toxicity among these toxins [39,82]. However, most publications have demonstrated that 3-AcDON has a relatively low toxicity. The comparison of 15-AcDON cytotoxicity data with those of DON has shown that no statistically significant differences occur between the cytotoxicity of the two compounds [23,25,31,43,82].

One of the theories concerning this issue is a possible increase in toxicity, which can be attributed to esterification at the C-15 position, and its decrease, which can be attributed to acetylation at the C-3 position. To a certain extent, the differences in the results obtained by the authors can be explained by the fact that the correlation between the chemical structure and toxicity is affected by various interactions, which depend on the cell line used [43,83,84]. DON binds to a ribosome through three hydrogen bonds, while its acetylated derivatives form two bonds: one is formed within the epoxy group and the other within the hydroxyl group. The presence of the acetylated group in 15-AcDON and 3-AcDON influences the binding strength of the toxin-ribosome complex. The acetylated group at position 15 allows an additional stabilising hydrophobic bond, whereas the same group at position 3 induces the emergence of stabilising van der Waals interactions [22,27,85].

Breokard and colleagues carried out some in-depth studies, including flow cytometry analysis. An epithelial cell line from the intestines of newborn IPEC-J2 piglets was used to compare the toxicity of DON and its derivatives. The choice of this cell line was supported by the high susceptibility of pigs to DON, and these cells were neither transformed nor cancerous. The surfaces of the cells, grown within two groups (varied and proliferative) in a monolayer, were exposed to different concentrations of the tested compounds. After the results were reviewed, the following toxicity ranking was drawn: 15AcDON ≈ DON > 3AcDON >> DON-3G [25]. This ranking is consistent with the report of a study that relates the toxicity of these compounds to the presence of free hydroxyl groups, which influence the affinity to ribosomes, at the C3 carbon. These studies also demonstrated that DON derivatives had stronger toxic effect on proliferative cells. The increased sensitivity of the cells to DON and its derivatives is associated with translation inhibition and a change in the activity of metabolically vital enzymes [25,43]. Moreover, negligible or no DON-3G toxicity has been demonstrated in IPEC-J2, Caco-2, and GES-1 cell lines [31,82,86,87]. The toxicity of DOM-1 has recently been explored in IPEC-J2 cell line, where lysosomal activity, total protein content, cell membrane integrity, metabolic activity, ATP level, and the ability to induce apoptosis were determined. All studies indicated that DOM-1 had no statistically significant impact on the tested cells within the concentration range used (0–100 µM). Additionally, studies on Caco-2 cells showed that DOM-1 has no cytotoxic effects [82,88].

DON, 3-AcDON, and 15-AcDON can cause lipid peroxidation in the intracellular environment. The exposure of HepG2 cells to these toxins causes an increase in the concentration of malondialdehyde (MDA), a marker of lipid peroxidation. Similarly, statistically significant differences in the levels of reactive oxygen species (ROS) produced were observed only upon exposure to 15-AcDON. These results imply that the cytotoxicity of DON and its acetylated derivatives is, to a significant degree, independent of oxidative stress [30]. However, this phenomenon is more sophisticated and may depend on the type of exposed cells. Literature data available on the subject lack consistency and often lead to conflicting conclusions. For instance, with GES-1 cell line, oxidative stress induction was observed under the influence of DON and 15-AcDON. This stress is accompanied by increased ROS, decreased ATP levels, and mitochondrial respiration impairments manifested by NAD^+^/NADH imbalance [41]. DON and its acetylated derivatives are also likely to disturb the cell cycle. In the case of HepG2 cells, exposure to these toxins caused significant alterations in cell content at all the phases of the cell cycle, compared with the control sample. Similarly, increased numbers of cells were arrested upon exposure to 15-AcDON and DON at the G0/G1 and G2/M phases, which was the most significant cytotoxic effect observed for these compounds. Cell arrest at the G2/M phase implies that DON and 15-AcDON may induce DNA damage. During this phase, the cells pass the control point of the cell cycle. This is based on the activity of kinases, which arrest the cell cycle in response to DNA damage to prevent the multiplication of incorrect genetic information. In the case of 3-AcDON, significant induction of micronuclei formation was observed. According to the literature, this phenomenon is related to the ability to induce genotoxicity and cell cycle disruption [39,40]. Proapoptotic activity has also been demonstrated for DON and 15-AcDON in GES-1 cells. These compounds can induce apoptosis by activating the mitogene-activated kinases (MAPK) p38 and JNK, and inhibiting the ERK1/2 kinases. This mechanism of DON-induced apoptosis was confirmed in porcine hippocampal nerve cells [41,42]. Additionally, DON and 15-AcDON can significantly influence cell metabolism by altering the concentration of metabolites, such as nicotinic acid, niacinamide, sphynganine, adenine, serotonin, taurine, adenosine, phosphatidic and hydroxyphenyllactic acids, and glutamine. These metabolites are essential for oxidative phosphorylation processes and for maintaining metabolic balance. Changes in the levels of the metabolites can lead to proliferative alterations. However, similar studies have not been conducted for 3-AcDON, and hence, it cannot be determined if this compound has the same properties [41].

Recently, efforts have been made to evaluate changes in the transcriptome of cells exposed to DON, 15-AcDON, and 3-AcDON. The tests showed the impact of each of these toxins on the transcription of over 2000 genes as they disturb signalling routes and processes, such as replication, DNA repair mechanisms, and cell cycle. Some relevant alterations include an increased activity level of ATM kinase, which implies that DNA damage occurred in cells exposed to the tested toxins, resulting in cell cycle arrest. The mechanism of cell cycle inhibition through ATM kinase involves indirect p53 protein activation, which is activated by the cyclin-dependent kinase inhibitor, consequently making the formation of the CDK2-cyclin E complex impossible. This complex is critical for cells to reach the prereplicative state and enter the S phase. The increased expression level of sestrins genes associated with antioxidant defence implies that the aforementioned DNA damage may have been caused by oxidative stress [43].

In vitro research aimed at evaluating the cytotoxicity of DON metabolites carried out in recent years has focused on determining and comparing their IC_50_ values. Stomach, intestine, and liver cell lines are usually used for this purpose, as these organs have the highest exposure to foodborne toxins. Acetylated DON derivatives are the most toxic metabolites of DON. However, no studies have assessed the cytotoxicity of DON metabolites using cell lines representing other internal organs and systems, including the nervous system. Additionally, no toxicological research has been carried out on newly discovered plant metabolites, such as DON-glutathione (DON-GSH) or DON-3-sulphate and DON-15-sulphate. Limited sources have linked DON and its acetylated derivatives to lipid peroxidation and oxidative stress; however, the available reports are controversial. There are conflicting reports on the ability of DON and 15-AcDON to induce the generation of ROS, which can damage DNA. The possible genotoxicity of acetylated derivatives of DON is indicated by cell cycle impairments observed upon exposure and by the increase in the expression of genes associated with antioxidant defence. However, this has only been reported by one study, and hence, further investigation should be carried out.

### 3.2. Cytotoxicity in In Vivo Systems

An adverse feature of 3-AcDON and 15-AcDON is that these compounds are absorbed in the intestines at levels two- and four-fold higher, respectively, than DON. This can be attributed to their less polar structure and higher capability for passive, non-ionic diffusion [27]. Previous studies on animals reported low in vivo persistence of acetylated forms of DON. It was observed that after the oral administration of 3-AcDON and 15-AcDON, pigs only had traces of these compounds in their blood. Both compounds were capable of deacetylation to DON in the gastrointestinal tract (GI) as a result of interactions with bacteria [27]. In the case of chickens, de-epoxidation reactions of 3-AcDON and 15-AcDON to DOM-1 were also observed. Intensive deacetylation also occurred within the circulatory system, which was confirmed by intravenous administration of these compounds. During the biotransformation phase II, DON couples with glucuronide, although some organisms, such as rats, are able to influence glucuronidation of the acetylated forms [27]. In some studies involving pigs, attempts were made to determine the toxicokinetics of DON-3G. Upon oral administration, this compound was not detected in the plasma, possibly due to its hydrolysis in the GI tract. This was evident by the presence of DON in the plasma of blood drawn from the portal vein of animals from the test group. Following the intravenous administration of DON-3G in pigs, DON was not detected in the plasma, thus proving that this compound does not undergo hydrolysis in the circulatory system [86,89,90]. In broiler chickens, the hydrolysis of DON-3G was not observed either after intravenous or oral administration. This observation implies that the hydrolysis of DON-3G in the GI depends on the composition of the intestinal microflora [86,90]. Following the oral administration of DOM-1 in pigs, the characteristic effects of DON were not observed. The animals had normal body weight and no pathological changes in the intestines or liver. Furthermore, no behavioural disorder or vomiting was observed throughout the experiment. However, similar to DON, DOM-1 had an impact on the expression of E-cadherin in the jejunum by significantly reducing its level [37]. However, the possible induction of E-cadherin expression in pigs exposed to DOM-1 is controversial. The results of recent studies on the subject seem to contradict these observations. Nevertheless, the results related to the other aspects implied that DOM-1 did not induce toxic effects in the pig model. Bracarense et al. used the same method to study the possible toxicity of 3-epi-deoxynivalenol (3-epi-DON), and they did not observe any changes compared with the control samples [38]. A summary of the described in vivo studies is presented in Table 2.

### 3.3. Immunotoxicity

Recent research has revealed that DON can induce multiple toxic effects in the immunological system. However, the available literature on DON metabolites is considerably more modest [91,92,93]. In Caco-2 cell line, DON and its acetylated forms both induced the expression of pro-inflammatory CXCL8 (IL-8) cytokines to a similar degree. This ability was not observed with DON-3G or DOM-1. This cytokine is vital for the toxicological assessment of xenobiotics from the gastrointestinal tract as it plays a major role in the process of local intestinal inflammation [82]. DOM-1 administered to pigs did not have any considerable influence on the expression of cytokines IL1-α, IL6, IL10, IL17-α, and TNF-α; moreover, it did not induce apoptosis within the spleen or lymph gland cells. The lack of a major impact of this toxin on the expression of pro-inflammatory cytokines was seemingly confirmed by studies involving intestinal explants [37,38]. DOM-1 displayed immunomodulatory properties similar to those of DON. It increased a specific immunologic response measured as the quantity of antibodies against a vaccine and cell proliferation in the lymph glands. This implies that DOM-1 can be an adjuvant that can prolong the exposure time of antigens in the body, amplifying the immunological response [37]. Furthermore, DOM-1 activates eukaryotic translation initiation factor 2 alpha kinase 2 (EIF2AK2). DON has this property due to its ability to bind to ribosomal RNA, which induces translation inhibition. DOM-1 also amplifies the transcription of genes that encode endoplasmic reticulum (ER) stress-related proteins, ATF4 and PRKRA [94]. The available information on DON-3G immunotoxicity is limited. Explants from pig intestines have recently been used to study the impact of exposure to this toxin. DON-3G did not alter the expression of the assayed pro-inflammatory cytokines, including IL-1, IL-1β, IL-8, IL-17A, IL-22, and TNF-α. No significant changes were observed in the expression of genes associated with inflammation, including NFk-B complex genes [87]. Similarly, a recent study that assessed changes in the expression of pro-inflammatory cytokines reported that 3-epi-DON had no effect on the immunological system [38].

### 3.4. Interactions

Most of the studies that assessed the toxicity of mycotoxins and their derivatives have only considered exposure to a single compound. Fungal contamination leads to the occurrence of different mycotoxins and their derivatives in food; hence, assessing the toxicological interactions that can occur in such instances is necessary. Concurrent exposure to multiple toxins can lead to toxic effects, referred to as additive, synergistic, and antagonistic effects [26,31]. From a toxicological perspective, the synergistic effect is highly adverse, as it defines the case where exposure to a mixture of toxins induces stronger toxic effects than the combined toxic effects produced by individual components of this mixture (additive effect) [26,31]. In 2013, a cell study with Caco-2 cell line demonstrated that synergism is the major type of interaction that occurs in cells following exposure to group B trichothecenes. This synergism was observed when the toxins were used at concentrations that resulted in a reduction in the proliferation of cells tested within the range of 10–40%. Higher concentrations had characteristic additive or nearly additive effects [95]. In IPEC-1 cell line, DON-15-AcDON and 3-AcDON-15AcDON showed synergistic effect following the exposure of the cells to high toxin concentrations, which reduced the proliferation of the cells by 80%. However, the DON-3-AcDON mixture exhibited an antagonistic effect, which decreased in intensity with increasing concentrations, until it was overtaken by the synergism at higher concentrations [26]. Synergism has also recently been reported for DON+15-AcDON and 3-Ac-DON+15-AcDON mixtures in GES-1 and HepG-2 cells, respectively [31,96]. This interaction can possibly be attributed to the different affinities of toxins to efflux transporters, such as P-gp and MRP2. The synergistic effect is caused by the transporters being saturated by the weaker toxin, resulting in increased accumulation of the more toxic component in the mixture [26]. This theory is consistent with the higher concentrations of 3-AcDON, compared with that of 15-AcDON, in a culture medium of HepG-2 cells [96]. It has also been reported that the synergy of DON+3-AcDON, DON+15-AcDON, and 3-AcDON+15-AcDON mixtures also influences the induction of ROS and lipid peroxidation, with 3-AcDON+15-AcDON mixture having the most pronounced effects [30]. Additionally, this mixture showed the highest synergy in the induction of micronucleus formation [39]. A summary of the described in vitro studies is presented in Table 3.

## 4. Modified Forms of T-2 and HT-2

### 4.1. Cytotoxicity

Cytotoxicity assay with human liver carcinoma HepG2 cells showed that the cytotoxicity ranking of the toxins were HT-2 ≈ T-2 > NEO > T-2 Triol > T-2 tetraol, in reference to the IC_50_ values. Similar results were observed for the two cytotoxicity tests adopted in the study, which involved the analysis of mitochondrial dehydrogenase activity (MTT) and the total protein content (PC), and this increases the credibility of the aforementioned ranking [98]. A relatively low toxicity of T-2 triol and T-2 tetraol was observed in the study. The comparable toxicity of T-2 and HT-2 may be a result of the rapid and efficient transformation of T-2 into HT-2. It has been shown that the efficacy of T-2 biotransformation into HT-2 in the HepG2 cell line can reach up to 94% [32,98]. A slightly differing toxicity ranking was determined in a study using porcine Leydig cells: T-2 > HT-2 > T-2 triol > NEO > T-2 tetraol [33]. The lower toxicity of T-2 metabolites, compared with that of their parent toxin, may be caused by the presence of additional hydroxyl groups formed following the hydrolysis of ester bonds. Wu et al. (2010) reported that T-2 triol and T-2 tetraol are less toxic than T-2 due to acetoxylated groups substituted with hydroxyl groups at the C-15 position. These studies confirm the hypothesis that hydrolysis and hydroxylation are the major mechanisms associated with the detoxification of group A trichothecenes [32,33,68,69]. The cytotoxic effects of T-2 and HT-2 on the HepG2, Caco-2, RAW 264.7, and HEK 293T (human embryonic kidney cells) cell lines were assayed using resazurin. This assay indicated the special sensitivity of the HEK 293T cell line to these compounds [34]. Studies that comprehensively compare the cytotoxicity of T-2 and HT-2 toxin metabolites are limited. The results of these studies vary according to the adopted methodologies and the selected cell line, thus leading to divergent conclusions. Nonetheless, regardless of the aforementioned factors, the authors point out a higher cytotoxicity of T-2, compared with that of its metabolites [32,33,34]. Prospective research should primarily focus on assessing the cytotoxicity of plant metabolites of T-2 and HT-2 on stomach, intestinal, and liver cells. At present, to the best of our knowledge, no study has reported the toxicity of T-2 and HT-2 glucosides. Further studies are necessary to evaluate the toxicity of human metabolites of T-2 and HT-2, particularly with reference to their possible neurotoxicity and nephrotoxicity.

### 4.2. Studies in In Vivo Systems

Cross-over trials in animals have been carried out with the intraperitoneal and oral administration of T-2 toxin and T-2-3-α-glucoside (T-2-α-Glc) in broiler chickens. Plasma concentrations of T-2, T-2-α-Glc, and their major metabolites were quantitatively determined using fluid chromatography. T-2 triol was not detected, while only a trace amount of HT-2 was detected in the plasma samples. No adverse effects were observed in any of the animals during the study, regardless of the route of toxin administration. Following oral administration, T-2-α-Glc was absorbed five-fold faster than T-2. Such a significant difference in intestinal absorption can be explained by the possible increased intestinal absorptive efficacy caused by glycosylation [99]. These results imply that the assumption of equal bioavailability of free and modified mycotoxins is not correct. Extracts of T-2 metabolites derived from shrimp fed with T-2 were obtained in the study by Zhanrui et al. (2017). The metabolites present in the extract were not identified, but the extracts contained no parent toxin. These extracts caused multiple pathological changes, such as liver damage, anaemia, and reduced bone marrow-forming activity. Furthermore, blood tests showed reduced counts of erythrocyte and platelet counts with increased neutrophil count [100]. Anorexia, which is a defensive response against food poisoning, is typical of trichothecene toxicity. Reports that describe possible mechanisms through which trichothecenes cause anorexia and compare the efficacy of anorexia induction by T-2 and its metabolites have recently been published [101,102,103]. The gut satiety hormones, cholecystokinin (CCK) and glucagon-like peptide-1(GLP-1), are vital for appetite regulation. They are secreted by enteroendocrine cells of the intestines and within the central nervous system and influence the expression of anorexigenic factors in the hypothalamus. Increased concentrations of the hormones were observed in mice following the administration of T-2, HT-2, and NEO. Increase in the concentration of CCK was more significant, which implied that its role in anorexia is more important. However, some cases of anorexia occurring prior to the rise in the levels of CCK and GLP-1 have been reported, which suggests that other factors are involved in the process [101]. Substance P (SP) and serotonin also known as 5-hydroxytryptamine (5-HT) are neurotransmitters produced in the CNS and GI tract by enterochromaffin cells. They regulate the expression of proopiomelanocortin and cocaine-and amphetamine-regulated transcript within the hypothalamus neurons, thus affecting the perception of hunger [104,105,106]. Studies on mice have demonstrated that T-2, HT-2, and NEO-induced anorexia are associated with 5-HT and SP. Both intraperitoneal and oral administration of these toxins to mice induced anorexia and caused significant increase in the levels of 5-HT and SP in the plasma. T-2 and HT-2 displayed slightly increased ability to induce 5-HT and SP expression, compared with NEO. All the aforementioned toxins greatly inhibited food intake, and simultaneously, the anorectic effect strongly correlated with the concentration of the tested neurotransmitters. Some cases, in which anorexia occurred before the increase in the concentrations of 5-HT and SP or in which it continued even after the levels of these neurotransmitters dropped, were observed [102]. The role of YY neuropeptide (PYY) may be critical in the induction of anorexia mediated by T-2 metabolites in mammals. It was noted that PYY concentration in minks exposed to diacetoxyscirpenol (a toxin from the trichothecene group) increased 30 min after oral or intraperitoneal toxin administration [107]. No analogous tests were performed for T-2 and its metabolites. In mice exposed to T-2, HT-2, and NEO intraperitoneally and orally, the ‘no observed adverse effect level’ (NOAEL) and ‘lowest observed adverse effect level’ (LOAEL) were determined. The NOAELs obtained for both oral and intraperitoneal administration of T-2, HT-2, or NEO did not vary and was 0.01 mg/kg body weight. Similarly, the LOAEL dose for T-2, HT-2, and NEO did not vary and was 0.1 mg/kg BW for both oral and intraperitoneal administration. Moreover, there was no significant difference in the intensity of the anorectic effect of T-2, HT-2, and NEO [103].

### 4.3. Immunotoxicity

Free T-2 toxin significantly affects the functionality of immunological system routes, but publications describing the impact of its metabolites on these routes are very limited [71,108]. Wang X. et al. aimed at assessing the immunotoxicity of T-2-glucuronide (T-2-GlcA) and free T-2 and the impact on critical signal routes in RAW264.7 cell line (murine macrophages). In the same experiment, the toxicity of unidentified T-2 metabolites (mT-2) in extracts derived from T-2-fed shrimps was also determined. The extracts did not contain free T-2; thus, its influence on the tested parameters was excluded. It was found that mT-2 and T-2-GlcA increased the expression of the cytokines IL-6, IL-1β, and TNF-α. The increase in the concentrations of IL-1β and TNF-α increase was negligible, implying that these compounds activated the JAK/STAT (Janus kinases/Signal Transducers and Activators of Transcription) pathway (main cytokine receptor signalling pathway) through IL-6. T-2 toxin induced the expression of IL-6 and TNF-α to a considerably greater degree that by mT-2 and T-2-GlcA, which confirms their relatively lower immunotoxicity. It was observed that mT-2 and T-2-GlcA increased the expression of JAK1, JAK2, and JAK3 along with STAT1, STAT2, and STAT3. The highest increase in expression was observed for STAT2, which was influenced by mT-2. In contrast, a considerable increase in the phosphorylation of STAT3 and JAK3 exposed to mT-2 and JAK2 exposed to T-2-GluA was observed. Moreover, mT-2 and T-2-GluA increased the expression of proteins from the SOCS family (SOCS1, SOCS3, and CIS), which are responsible for negative feedback at increased concentrations of pro-inflammatory cytokines [109].

### 4.4. Interactions

In studies involving HepG2 cell line, antagonistic effect was reported for T-2+HT-2, T-2 triol+HT-2, T-2 tetraol+HT-2, T-2+T-2 tetraol, and T-2 triol+T-2 tetraol mixtures at lower concentrations, while additive effect was observed at higher concentrations. An antagonistic effect independent of concentration was observed for T-2 triol+T-2, T-2 triol+ NEO, T-2+NEO, NEO+HT-2, and T-2 tetraol+NEO mixtures, which possibly stem from the less toxic toxins that block their shared binding sites. Due to differences in chemical structure (presence of steric hindrance), NEO, T-2 triol, and T-2 tetraol supposedly showed greater receptor affinity than T-2 and HT-2 [32]. These results are not consistent with the synergistic effects described for T-2+HT-2, T-2+NEO, and HT-2+NEO mixtures in porcine Leydig cells; however, an antagonistic effect was also observed with higher toxin concentrations [33].

### 4.5. Bioinformatic Evaluation of Toxicity

In silico toxicity analysis is becoming increasingly significant, as this approach involves reduced cost and time. Numerous programs are used for this analysis, and they share the features of the possibility of ADME prediction (absorption, distribution, metabolism, and excretion). These methods use both the structure–activity relationship and experimental and literature data for this purpose [110,111]. Although a bioinformatic toxicity evaluation of DON metabolites has rarely been reported in the literature, it has been used extensively for the toxicity evaluation of T-2 metabolites. T-2 metabolites (NEO, T-2 triol, and T-2 tetraol) were analysed using AdmetSAR software. This tool uses physicochemical properties, such as molecular weight, l-octanol/water partition coefficient logarithm, and the presence of donor and acceptor hydrogen bonds to determine the affinities of specific proteins [112]. NEO, T-2 triol, and tetraol showed strong affinity for the P-gp transporter. This property indicates low bioavailability, as this transporter is responsible for the return delivery of xenobiotics to the intestinal lumen. The role of P-gp also involves the prevention of toxins from penetrating vital tissues and internal organs, participation in toxin metabolism by acting together with the CYP450 3A4 cytochrome, and acceleration of toxin excretion by acting on renal tubules and bile ducts. A high affinity for P-gp usually means a high metabolic efficacy of CYP450 3A4, as they have similar substrate specificities. T-2, NEO, T-2 triol, and T-2 tetraol are also able to inhibit the transporter activity of OATP1b1 and OATP1b3 hepatocytes. In living organisms, this attribute can lead to inhibited proliferation, hepatotoxicity, and cholestasis of hepatocytes [32]. Crystallographic analyses enabled the precise determination of the trichothecene binding site within the ribosome. This site is characterised by hydrophobicity at the central point and peripheral hydrophilicity. The ability of mycotoxin molecules to act as hydrogen bond donors is of key importance in the formation of toxin-ribosome complexes. The use of bioinformatics enabled the evaluation of the affinity of specific mycotoxins to the binding site and their toxic potentials [113]. The authors found that substituents at position 3 (T-2-3-glucuronide, T-2-3-α-glucoside, and T-2-3-β-glucoside) led to spherical interactions impeding binding with a ribosome; hence, these metabolites may not be highly toxic [113]. It is worth noting that in the study, the affinity ranking for the modified forms of T-2 was as follows: 19-hydroxy-T-2 ≈ 20-hydroxy-T-2 ≈ 15-deacetyl-T-2 > NEO > T-2 > T-2 triol ≈ T-2 tetraol > HT-2 > T-2-3-α-glucoside > T-2-3-β-glucoside ≈ T-2-3-glucuronide [113]. Nevertheless, in addition to ribosomal affinity, more factors should be considered when assessing the toxicity of mycotoxins. The distribution and excretion of mycotoxins are of key importance from the perspective of absorption kinetics. Combined insights into all the aforementioned phenomena would facilitate a reliable review of the toxicity of the investigated compounds. The evaluation of the ability of mycotoxins to bind plasmatic proteins is significant for toxicity assessment, as the resultant complexes reduce toxin bioavailability. It has been reported that T-2 and HT-2 have a similar probability of forming complexes, and these compounds do not differ in terms of their ability to bind to the oestrogen receptor [34].

### 4.6. Metabolism vs. Toxicity

The toxicity of T-2 metabolites also largely depends on their metabolism and fate in an organism. T-2 derivatives containing a glucoside group neither bind with a ribosome nor activate MAPKs. However, there are no publications on this subject, and the hypothesis is based on conclusions from a study on DON-3G [71]. In the case of T-2-3-α-glucoside (T-2-α-Glc) and T-2-3-β-glucoside (T-2-β-Glc), no structural alterations were found upon contact with the saliva or intestinal digestive juices [114]. However, these compounds can reproduce their parent aglycones in the large intestine, following fermentation by bacterial flora. The faecal bacteria transformed the compounds with the following efficacy: T-2-α-Glc to T-2 (13%) and HT-2 (30%), and T-2-β-Glc to T-2 (58%) and HT-2 (12%) [115]. However, in vivo, the hydrolysis of glycosylated T-2 and HT-2 derivatives can proceed so slowly that obtaining the above efficacy levels becomes impossible. Furthermore, this process seems to be highly dependent on the composition of bacterial flora, as other authors experimentally obtained a 100% conversion rate to HT-2, following a 24-h incubation of T-2-β-Glc on human faeces samples [114,116]. This phenomenon has also been studied with the use of porcine bacterial flora in an ex vivo model using porcine jejunum, and rapid and efficient hydrolysis of T-2-Glc and HT-2-Glc to T-2 and HT-2, respectively, was also achieved. In this case, further transformation of the resultant aglycones to T-2 triols and HT-2 was also observed [117]. In studies on Caco-2/TC7 (small intestine cells), T-2-Glc did not show the ability to move both to the basolateral compartment and through the epithelium monolayers, unlike T-2 and HT-2. Moreover, the epithelial cells were unable to hydrolyze T-2-Glc. It has been postulated that these compounds could hydrolyse within the circulatory system [71,114], but there are currently no publications on this phenomenon.

## 5. Modified Forms of ZEN

### 5.1. Cytotoxicity

ZEN is commonly claimed to have cytotoxic properties. It has been linked to oxidative stress induction, apoptosis, DNA damage, and cell cycle arrest [118,119,120,121]. As the liver and intestines have the highest exposure to ZEN and its metabolites, most studies have focused on assessing their cytotoxicity using cell lines derived from these organs. However, the obtained results tended to vary with the adopted methodologies and cell lines. Variations have also been observed in the IC_50_ values reported by different studies involving same compounds and cell lines. Similarly, cytotoxicity rankings and conclusions drawn on the bases of these rankings are often different. A higher cytotoxicity of β-ZOL, in comparison with that of ZEN and α-ZOL, has been reported by most studies in the literature. β-ZOL was the most cytotoxic in HepG2 (liver cells), Caco-2 (small intestine cells), RAW264.7, and SH-SY5Y (bone marrow) cells [35,36,48,122,123]. The potentially lower membrane permeability of α-ZOL, compared with that of β-ZOL, can affect its toxicity. In studies conducted using SH-SY5Y bone marrow cells, the level of α-ZOL was higher than those of ZEN and β-ZOL in the culture medium, which implies that α-ZOL is not easily absorbed by cells [48]. The neutral red assay was conducted using HepG2 cells, and the results showed a significant reduction in cellular longevity following the 72-h exposure of cells to ZEN, α-ZOL, and β-ZOL. The highest cytotoxity level was displayed by β-ZOL, with the IC_50_ value at 13.1 μM, compared with 39.7 μM and 119 µM for ZEN and α-ZOL, respectively [35]. However, these results are not consistent with the data obtained on the basis of cell metabolic activity (MTT assay) using cells of the same line, where α-ZOL showed higher cytotoxity than ZEN (IC_50_ of 131.40 μM and 143.35 μM, respectively). The large differences between the IC_50_ values obtained in the two analyses occurred as a result of different incubation times, namely 72 or 24 h. However, it is noteworthy that ZEN showed different toxicity, compared with α-ZOL, in both experiments [36]. The studies by E. Tatay et al. also pointed to a higher toxicity of α-ZOL, the IC_50_ values of which were lower than those of β-ZOL and ZEN, regardless of the incubation time, which were 24, 48, and 72 h [124].

To assess the effect of ZEN and its metabolites on intestinal cells, a Caco-2 cell line was selected for the test. Based on the results, the following toxicity ranking was suggested: ZEN > β-ZOL > α-ZOL. MDA level, DNA fragmentation, and caspase 3 activity were determined to compare the mechanisms of molecular cytotoxicity. ZEN showed the highest ability to induce MDA formation. The levels of its metabolites were noticeably lower, with a slight advantage for β-ZOL. MDA is an oxidative stress marker, which is the final product of lipid peroxidation. The effects of lipid peroxidation include pathological alterations to molecular membrane and metabolism, which leads to cytotoxicity [122,125]. In addition to lipid peroxidation, oxidative stress can result in DNA damage, which can lead to apoptosis. Both ZEN and its metabolites have a similar ability to induce DNA fragmentation, the intensity of which depended on their concentrations. Regardless of the concentrations used, ZEN activated caspase 3 more intensively than α-ZOL and β-ZOL. Cell exposure to ZEN also decreased the level of anti-apoptotic Bcl-2 protein. Caspase 3 plays a vital role during apoptosis, as it participates in DNA fragmentation, chromatin condensation, and the formation of apoptotic bodies. However, it cannot be clearly stated that the metabolised ZEN has a reduced capacity to induce apoptosis, as this process can take place in a manner partly independent of caspase 3 [122,126,127].

However, the cytotoxic mechanisms of ZEN and its metabolites vary in different cell lines. This is related to disturbances caused by different biochemical pathways in various tissues [128]. In RAW264.7 cell line (murine macrophages), β-ZOL displayed significantly higher cytotoxicity than α-ZOL. Using flow cytometry, both metabolites caused cell death through apoptosis to a greater extent than through necrosis. The mechanism of apoptosis induced by these metabolites seems to be independent of caspases and occurs under the influence of mitochondrial stress. A noticeable decrease in the mitochondrial membrane potential (MMP) was observed following exposure of cells to α-ZOL and β-ZOL. Reduced MMP levels result in the release of apoptogenic proteins from the mitochondria. The caspase-independent induction of apoptosis by ZEN metabolites is supported by the fact that caspase inhibitors did not significantly affect cell longevity as well as by the observed release of proapoptotic AIF protein from the mitochondria under the influence of the tested toxins, the intensity of which was dependent on their concentrations [123].

It has been recently reported that α-ZOL and β-ZOL induce endoplasmic reticulum stress. This process is markedly associated with cytotoxicity, as it can lead to mitochondria-dependent cell apoptosis. Increased levels of the markers of endoplasmic reticulum stress, such as GRP78 and GADD34 proteins, were observed upon the exposure of HCT116 cells (colon cells) to α-ZOL and β-ZOL. The concentration of the C/EBP homologous protein (CHOP) transcription factor—which is the main component of apoptosis induced by endoplasmic reticulum stress—increased by nearly 10-fold. The relationship between cell apoptosis and mitochondria is supported by the significantly lower mitochondrial transmembrane potential, as well as the relationship between apoptosis and the presence of BAX and BAK proteins. Interestingly, the tested cells showed significantly increased caspase-3 activity. This contradicts the independence of apoptosis induction on ZEN metabolites described earlier and should be the subject of further studies [129]. Data regarding the cytotoxicity of plant-derived ZEN metabolites are limited. Following exposure to ZEN-14G, no significant reduction in the longevity of MCF7 cells was observed at a concentration of 1 μM. Similarly, this compound did not show cytotoxicity towards Caco-2 cells at concentrations of 20 and 40 μM [130,131]. To date, no studies have assessed the cytotoxicity of plant-derived ZEN metabolites at various concentrations. This knowledge is still missing in the context of fungi-derived ZEN metabolites (e.g., zearalenone-14-sulphate, ZEN-14S) [65].

### 5.2. Studies in the In Vivo Systems

The toxicokinetic properties of ZEN and its metabolites were compared in pig studies. No conversion was observed following intravenous administration of α-ZOL and β-ZOL. These compounds were transformed into ZEN with low efficacy; however, a higher conversion efficacy into ZEN (approximately 20%) was displayed by ZEN-14G. Intravenous administration of these toxins in rats also showed a higher conversion efficacy of the glucoside derivatives of ZEN to the parent compound. The conversion rate of α-ZOL-14G to ZEN was five-fold higher than the analogous conversion rate for α-ZOL. These reactions occur under the influence of blood esterases and liver enzymes [132,133]. However, it is worth noting that no metabolites were detected in the blood of the pigs after the intravenous administration of ZEN-14S. This indicated its rapid elimination instead of the hydrolysis reaction, which occurred in the case of ZEN-14G. In pigs, ZEN displayed a longer half-life but lower bioavailability, compared with its modified forms. The longer half-life can be attributed to the lower polarity of ZEN in comparison with its modified forms. Another explanation involves probable differences in affinity to plasma proteins. Following the oral administration of ZEN14-G and ZEN-14S, significant differences were observed in the time elapsed until their metabolites were detected in the blood, with a much longer delay observed for ZEN-14S. Such a considerable delay implies that ZEN-14S mostly undergoes hydrolysis in the distal section of the intestine, whereas ZEN-14G hydrolyses earlier within the duodenum and jejunum. Moreover, the hydrolysis of ZEN-14S occurred within the stomach and small intestine. Following the oral administration of ZEN-14G and ZEN-14S, the compounds were not detected in the portal venous system, in contrast to α- and β-ZOL, which were found in small quantities. A few factors, such as low stomach pH, intestinal flora enzymes, and epithelial enzymes, play a role in the efficient hydrolysis of the aforementioned toxins. Following the oral and intravenous administration of a significant dose of ZEN-14G (500 μg/kg BW), traces of the compound were not detected in the urine. However, the urine contained ZEN at a significant concentration, which can be explained by the conversion of ZEN-14G to ZEN. A different experiment, using a considerably lower toxin dose (15.1 µg/kg BW for ZEN-14G and 12.5 µg/kg BW for ZEN-14S), reported that ZEN-14S was not present in urine following oral administration. In the study, the urine also contained ZEN, which indicated the hydrolysis of ZEN-14S [132,134,135]. Low bioavailability of α-ZOL and α-ZOL-14G was observed in rats, which can be explained by their efficient metabolism within the GI system [28,133]. The toxicity of ZEN-14S was compared with that of ZEN in a recent study using Caenorhabditis elegans grown on media containing the aforementioned mycotoxins. Although not widely used in the assessment of mycotoxin toxicity, this organism offers numerous benefits, the most important of which is its genetic similarity to mammals and a short development time. In vivo, ZEN-14S was reduced to α-/β-ZOL-14S, similar to what was observed with ZEN. Unlike ZEN, ZEN-14S did not decrease the longevity of the nematodes. ZEN-14S did not significantly influence the induction of oxidative and thermal stress. However, ZEN-14S and ZEN reduced the number of offspring of the tested nematodes at a similar rate. The underlying mechanism for this observation is unclear and should be the subject of further studies [136]. A summary of the described in vivo studies is presented in Table 4.

### 5.3. Immunotoxicity

Upon exposure of HepG2 cells to ZEN, α-ZOL, and β-ZOL, the expression of pro-inflammatory cytokines, such as IL-1β, IL-8, and TNF-α, reduced, with the highest effect observed for β-ZOL. The implied immunosuppressive activity of ZEN and its metabolites can have a significant adverse impact on health, leading to a weaker inflammatory response to pathogens and xenobiotics [35]. The ability of α-ZOL to reduce the proliferation of T cells was also demonstrated. This property can be attributed to its ability to induce apoptosis (indicated by higher caspase 3 level) and necrosis (indicated by the presence of LDH in the extracellular environment) in these cells. In T cells, α-ZOL also inhibited the transcription of cytokines IL-2 and IFNγ, which have pro-inflammatory properties [137]. Unfortunately, there are currently no studies describing the impact of other ZEN metabolites on T cells. The immunosuppressive activity of ZEN and its metabolites has also been confirmed by studies in pigs. ZEN, α-ZOL, β-ZOL, and ZAN significantly reduced the longevity of neutrophils (polymorphonuclear cells) and peripheral blood mononuclear cells (PBMC). It is currently impossible to clearly determine which ZEN metabolite is the most cytotoxic towards immune cells. For instance, the highest reduction in the lifespan of neutrophil was observed following exposure to ZAN, and the lowest was observed with ZEN. Moreover, in the case of PBMCs, ZAN was noticeably less toxic than ZEN. Furthermore, all the tested toxins significantly reduced the production of IgG, IgA, and IgM antibodies, and inhibited the expression of IL-8 in neutrophils and TNF-α in PBMCs at a similar rate [138,139].

### 5.4. Interactions

The co-occurrence of ZEN and its metabolites in cereal grains has been widely reported. The levels of the modified forms of ZEN in cereal products can be comparable to the toxin levels of their basic analogues [28,140]. The attempt to evaluate the impact of exposure to a mixture of ZEN and its metabolites has been made in recent years. The results of these studies imply that synergy can occur in the induction of toxic effects with some combinations of the tested toxins [35,48,128]. In studies involving HepG2 cell line, synergy was observed for the following mixtures: α-ZOL + β-ZOL, ZEN + α-ZOL, and ZEN + β-ZOL. This means that the IC_50_ values for these mixtures were lower than the sum of the IC_50_ values of their respective constituent mycotoxins. In a mixture of ZEN with α- or β-ZOL, a synergistic effect, which increased with elevated concentrations, was observed. Synergy is typical for these mixtures at higher concentrations with regard to the expression of pro-inflammatory cytokines [35]. The observed synergy in the cytotoxicity effects of α-ZOL + β-ZOL, ZEN + α-ZOL, and ZEN + β-ZOL has also been described in a study by Tatay et al. The synergism decreased with time, turned into an additive effect, and finally became antagonism. After 72 h of culture, antagonism was observed for all the aforementioned toxin mixtures. However, no probable explanation of this relationship was proposed in the study by Marin et al. Similar effects were not observed, despite using the same cell line, exposure time, or concentration range for the tested toxins [35,124]. Similar to the case of HepG-2 cells, synergism increased with increasing toxin concentrations in SH-SY5Y cells (neuroblastoma cells) exposed to α-ZOL + β-ZOL mixture [48]. Unfortunately, no studies have been conducted using other mixtures of ZEN and its metabolites for this cell line.

### 5.5. Estrogenic Activity

ZEN and its derivatives are structurally similar to oestrogen and are oestrogen receptor (ER) agonists. They have the ability to induce the expression of endogenous oestrogen-activated genes and induce a physiological response to in vivo oestrogen, including the modification of steroid metabolism and stimulation of oestrogen-dependent growth cells. Mycotoxins displaying this activity are also referred to as mycoestrogenes [141,142,143]. Excessive estrogenic activity can result in a series of diseases, including cancer (ovarian, breast, prostate, and testicular), obesity, sexual behaviour disorders, premature puberty in females, and decreased fertility in males [144]. The oestrogen activity of ZEN, α-ZOL, and β-ZOL was determined by evaluating their ability to induce the proliferation of breast cancer cells (MCF-7 line), expressed as the relative proliferative effect (RPE). A stable relationship with an increase in oestrogen concentration is typical for the MCF-7 cell line. The highest proliferation increase occurred upon exposure to α-ZOL, which was considerably higher than that of ZEN and β-ZOL. Exposure to higher toxin concentrations (18.75 and 25 µM) resulted in lower observed RPE values, which indicated cytotoxicity. The greatest decrease in proliferation was observed with α-ZOL, for which negative RPE values were recorded, and this was not observed with the other tested toxins. The hydroxyl group at the C6 position is supposedly responsible for the increased affinity of α-ZOL to oestrogen receptors (ERs). The obtained results imply that ZEN and β-ZOL can be classified as partial ER agonists and α-ZOL as an absolute agonist at concentrations ranging from 6.25 to 9.37 µM [141]. The oestrogen activity of ZEN and α-ZOL was also determined using a transfected MCF-7 cell line, which contained a luciferase gene coding plasmid, the transcription of which depended on the oestrogen concentration. In these studies, the xenoestrogenicity measured as transcriptional response was significantly higher for α-ZOL than for ZEN [145]. The conclusions drawn from the review of the available literature on ZEN and its metabolites allow for the following ranking of oestrogen activity: α-zearalenol > α-zearalanol ≈ cis-α-zearalenol > ZEN ≈ ZAN ≈ cis-zearalenone ≈ β-zearalanol ≈ cis-β-zearalenol > β-zearalenol [140]. Currently, the literature describing the estrogenic activity of plant-derived ZEN metabolites is very limited, but in studies on MCF-7 cell line, ZEN-14G was converted to xenoestrogenes, such as α-ZOL and β-ZOL [130]. The affinity of ZEN-14G to ERs, as measured by acellular assays, was 300-fold lower than that of ZEN. This observation was consistent with the considerably inhibited ER binding by ligands with a glucoside group, as described in the literature. Moreover, efficient ER activation upon exposure of the MCF-7 cell line to ZEN-14G was observed in cellular xenoestrogenicity assays. Unlike ZEN, ZEN-14G was not detected in the cellular lysates. Therefore, it can be concluded that the xenoestrogenicity of ZEN-14G is a result of its hydrolysis to compounds that show ER affinity [146,147].

### 5.6. Oxidative Activity

Oxidative stress is indicated as one of the causes of mycotoxin toxicity by a number of studies. It refers to a loss of balance between oxidants, for example, ROS and antioxidants, which results in damages to lipids, proteins, and nucleic acids. Oxidative stress occurs as a result of intracellular accumulation of ROS, which can be produced upon exposure to mycotoxins. The following enzymes act as a cellular defence against oxidative damage: catalase (CAT), superoxide dismutase (SOD), and glutathione peroxidase (GPx), the concentrations of which increase upon exposure to antioxidants [119,148,149]. The exposure of HepG2 cells to ZEN, α-ZOL, and β-ZOL resulted in a significant increase in ROS concentration, compared with that observed in the control samples. Only a slight difference in ROS concentrations, regardless of the concentrations of mycotoxins, was observed. This can be explained by an increase in the activities of antioxidant enzymes. Increased ROS levels are highly harmful, and their toxicity has been associated with metabolic oxidation, DNA mutations, as well as polymerase, DNA, and protein (including histones) damage. The tested cells showed significant dose-dependent DNA helix breakage caused by ZEN and its metabolites. α- and β-ZOL induced greater damage than ZEN, which implied lower genotoxicity of β-ZOL. α- and β-ZOL also increased the activity of SOD and GPx to a greater extent, which are enzymes involved in the neutralisation of ROS. Similarly, exposure to the tested mycotoxins resulted in decreased glutathione concentrations and CAT activity. Glutathione is the main component of non-enzymatic antioxidant defence, and its depletion can be attributed to consumption by Gpx for ROS oxidation. CAT catalyses the decomposition of hydrogen peroxide, but its activity can decrease or cease completely through oxidation at high concentrations. Such significant oxidative properties imply that ZEN and its metabolites are vital for cellular toxicity caused by ZEN and its metabolites [119]. A significant induction of oxidative stress was also observed during analogous research using CHO-K1 cell line (Chinese hamster ovary cells). ZEN, α-ZOL, and β-ZOL significantly increased intracellular ROS levels and indirectly induced DNA damage. Noticeably, more profound DNA damage was recorded upon exposure to α-ZOL and β-ZOL than ZEN, as was the case with the HepG2 cell line. Increased activities of SOD and GPx, interpreted as an adaptation of cells to higher oxidant quantities, as well as decreased CAT activity and glutathione concentrations, have been reported [149]. In a study in which RAW264.7 cells (murine macrophages) were exposed to α-ZOL and β-ZOL, these metabolites induced ROS production through the Fenton reaction. This indicates that oxidative stress caused by these toxins is mediated by the presence of hydroxyl radicals [123]. A summary of the described in vitro studies is presented in Table 5.

### 5.7. Induction of Epigenetic Alteration and Modulation of Gene Expression

Both ZEN and its modified forms, for example, α-ZOL and β-ZOL, can activate ERs. From an epigenetic perspective, this fact is of key importance, as ERs modulate the activities of many transcription factors and influence the expression of the components of many relevant biochemical pathways [141,150]. Furthermore, evidence that supports the ability of ZEN to induce alterations in the expression of nuclear receptors, DNA methylation, and histone modification is available [120,151]. α-ZOL increased DNA methylation, histone methylation, and acetylation in HepG2 cell line to an extent similar to that of ZEN. With polymerase chain reaction analysis, it was possible to relate these alterations to the enzymes responsible for the modifications. The expression of methylotransferases (DNMT1, EHMT2, PRMT6, and SETD8) and acetyltransferases (ESCO1, HAT1, and KAT2B) significantly increased, whereas that of histone deacetylases HDAC1 and HDAC3 decreased. DNA methylation and histone deacetylation are responsible for “gene silencing”, which is understood to inhibit their expression. These reactions play key roles, such as regulation of cell cycle, proliferation, and apoptosis [36,152]. Moreover, the toxicity of α-ZOL and ZEN also involved alterations in the expression of genes linked to key metabolic pathways and genes encoding nuclear receptors, such as PPARα, PPARγ, AhR, L-fabp, LXRα, LDLR, Glut2, HK2, and Akt1. As the IRS-1/PI3K/Akt signalling pathway and the PPAR receptor play an important role in the metabolism of glucose and lipids, as well as in insulin regulation, exposure to ZEN and α-ZOL can promote the occurrence of type 2 diabetes, atheromatosis, obesity, and other metabolic diseases [36,46,47,153,154].

### 5.8. Bioinformatic Evaluation of Toxicity

A comprehensive review of the toxicity of a compound also requires insight into the properties of its biotransformation products, which can also show toxic effects. Using the MetaTox tool, the products of ZEN, α-ZOL, and β-ZOL metabolism following O-glucuronidation, S-sulfurylation, and hydrolysis reactions were predicted. These products and the baseline toxins were analysed using SwissADME software to predict their absorption, distribution, metabolism, and excretion. ZEN, α-ZOL, and β-ZOL were established to be efficiently absorbed from the GI tract, but they are characterised by their low ability to cross the blood–brain barrier (approximately 30%). This ability rapidly increased as a result of sulfurylation (over 90%) and hydrolysis (over 70%). The possible toxicity of the tested compounds was evaluated using the PASS online software, which used the structure to assess the probability of activation of the respective biochemical pathways. ZEN, α-ZOL, and β-ZOL showed significant and comparable effects, leading to carcinogenesis, nephrotoxicity, and hepatotoxicity. However, compared with the others, ZEN potentially disrupted the functioning of the endocrine system to a considerably greater extent and was more genotoxic toward its metabolites. It was further demonstrated that the biotransformational reactions of ZEN and its metabolites significantly decreased its ability to induce apoptosis as a result of caspase 3 activation [43,45].

## 6. Conclusions

Recent toxicity research aimed at toxicity evaluation of DON, T-2, and ZEN metabolites conducted in recent years has mostly been carried out on cell lines representing the stomach, intestines, and liver. Studies have confirmed that acetylated DON derivatives are the most toxic metabolites of this compound, while the toxicity of T-2 and HT-2 metabolites was lower than that of their respective parent toxins. In vivo studies have also confirmed these observations. However, the toxicity of α-ZOL and β-ZOL remain unclear in relation to ZEN. Future in vitro toxicity research aimed at assessing the toxicity of the metabolites of the main *Fusarium* toxins should be carried out using cell lines representing internal organs other than those studied previously in order to assess probable neurotoxicity and nephrotoxicity. Evaluation of the toxicity of DON, T-2, and ZEN plant-derived metabolites is also important. Currently, no publications have described the cytotoxicity of the majority of these compounds. Toxicological data obtained from in vitro experiments should be analysed, taking into account the internal dose of the tested compounds. The following factors must be taken into account: bioavailability, rate of elimination, clearance, volume of distribution, maximum concentration, and rate of absorption. All of the above factors can be influenced by the toxicological interactions between mycotoxins. Recent studies have pointed out new findings on the toxicity of DON, T-2, and ZEN. The recently described induction of ROS generation by 15-AcDON is interesting, and hence the observation should be studied and described in broader terms, as it implies that this compound is characterised by genotoxicity and carcinogenicity. Based on in vivo research, anorectic effects induced in mice by T-2, HT-2, and NEO were described. This review highlights that the process involves gut satiety hormones CCK and GLP-1, as well as neurotransmitters 5-HT and SP. The role of the YY neuropeptide may be significant for this process, but no studies have demonstrated its association with T-2 and HT-2 metabolites. For ZEN metabolites, the possible mechanism of apoptosis induction by α-ZOL and β-ZOL was described. These toxins induce endoplasmic reticulum stress and mitochondrial stress, leading to the release of proapoptotic proteins. However, the role of caspase 3 still remains controversial in this process. Furthermore, the ability of α-ZOL to modify DNA through methylation and histone acetylation is highlighted. These modifications lead to alterations in gene expression, the products of which are components of crucial metabolic pathways. The disturbance of these pathways can contribute to the development of metabolic diseases, including type 2 diabetes. No analogous studies have been conducted on β-ZOL. Based on the results of in vivo studies, it may be concluded that acetylated DON derivatives, as well as T-2 and ZEN glucoside derivatives, efficiently hydrolyse parent toxins in the GI tract, and intestinal microflora plays a key role in this process. Further investigation of the phenomenon of intestinal absorption of T-2-Glc and HT-2-Glc is necessary, as previous in vitro and in vivo studies do not have clear conclusions.

## Figures and Tables

**Table 1:** The maximum content of *Fusarium* toxins in cereals in EU countries.

Unprocessed Cereals	Maximum Levels (μg/kg)	Source
**DON**		
Cereals other than durum wheat, oat, maize	1250	[7,8]
Oat, maize, durum wheat	1750	
**ZEN**		
Cereals other than maize	100	[7,8]
Maize	350	
**Sum of T-2 and HT-2**		
Oat	1000	[9]
Barley, maize	200	
Wheat, rye and other cereals	100	

**Table 2:** In vivo studies assessing toxicokinetics and toxicity of modified forms of DON.

Tested Animals	Tested Toxins	Exposure Type	Dose	Main Observations	Source
**Pigs**	DON DOM-1	Orally	0.5 nmol/kg BW for 21 days 1 mol/kg BW for 14 days	DOM-1 does not cause vomiting, body weight changes, or pathological changes in intestines and liver	[37]
	DON DOM-1 3-epi-DON	Orally	Unrestricted access to feed with toxin content of 3 mg/kg	DON and DOM-1, 3-epi-DON do not cause vomiting, body weight changes, or pathological changes in intestines and liver	[38]
	DON DON-3G	Orally and intravenously	55.7 µg/kg BW for DON-3G 36 µg/kg BW for DON	DON-3G does not undergo hydrolysis within the circulatory system and is not absorbed when administered orally	[89,90]
**Chickens** **Broilers**	DON DON-3G	Orally and intravenously	500 µg/kg BW for DON 774 µg/kg BW for DON-3G	DON-3G does not undergo hydrolysis within the circulatory system and gastrointestinal tract	[90]

**Table 3:** In vitro studies assessing the toxicity of the modified forms of DON.

Cell Line	Tested Toxins	Concentration Range and Exposure Time	Methodology	Main Conclusions	Source
**HEPG2**	DON 3-AcDON 15-AcDON	0–12.5 (µM) for 24 h	MTT	IC_50_ values (µM) were 4.3, 6.2, and 8.1 for DON, 3-AcDON, and 15-AcDON, respectively	[30]
			H_2_-DCFDA applied and fluorescence measured, TBARS applied and absorbance measured	Increase in ROS level was observed upon exposure to 15-AcDON. Lipid peroxidation was observed upon exposure to DON, 3-AcDON, and 15-AcDON	
		0–7.5 (µM) 3-AcDON, 15-AcDON 0–15 (µM) DON for 24 h	Neutral red assay	IC50 values (µM) were 3.90, 6.00, and 10.15 for 3-AcDON, 15-AcDON, and DON, respectively	[39]
		0–4.8 (µM) DON 0–3 (µM) 3-AcDON, 15-AcDON for 48 h	Flow cytometry	Toxins have the ability to disturb the cell cycle and induce micronucleus formation	
**GES-1**	DON 3-AcDON 15-AcDON DON-3G	0–3 (ppm) DON, 15-AcDON 0–12 (ppm) 3-AcDON, DON-3G for 24 h	Cell counting kit-8 (DOJINDO, Kumamoto, Japan)	The following toxicity ranking was proposed: DON >15-AcDON >>3-AcDON >DON-3G	[31]
	DON 15-AcDON	0–5 (μM) for 8 h	High-performance liquid chromatography-tandem high-resolution mass	Proving metabolic balance disturbances upon exposure to DON, 15-AcDON	[41]
			Western blot	Induction of apoptosis by DON and 15-AcDON was observed as a result of the activation of mitogene-activated kinases (MAPK) p38 and JNK and ERK1/2 kinases inhibition	
		5 (μM) for 30 min for the ROS assay 5 (μM) for 24 h for ATP and NAD+/NADH measurements	DCF-DA applied and fluorescence measured ATP Assay Kit NAD+/NADH Assay Kit	Increase in ROS level Decrease in ATP level and NAD+/NADH balance disturbances upon exposure to DON and 15-AcDON.	
**IPEC-J2**	DON 3-AcDON 15-AcDON DON-3G	0–20 µg/ml for 72 h	Flow cytometry	Following toxicity ranking: 15-AcDON ≈DON >3-AcDON >>DON-3G was drawn	[25]
	DON DOM-1	0–100 (µM) for 24, 48, and 72 h	NR, SRB, LDH, WST-1, MTT, CTG	DOM-1 showed no cytotoxicity	[88]
**CACO-2**	DON DON-3G	0–10 (µM) for 48 h	CellTiter-Glo Luminescent Cell Viability Assay (Promega, Madison, USA)	DON-3G showed no cytotoxicity	[87]
	DON 3-AcDON 15-AcDON DON-3G DOM-1	0–8.4 (µM) for 24 h for LDH assay for 4, 8, 12, and 24 h for TEER assays	LDH, TEER assay	Lower 3-AcDON cytotoxicity compared with that of 15-AcDON and DON DON-3G and DOM-1 showed no cytotoxicity	[82]
	DON 3-AcDON 15-AcDON	0–0.5 (µM) for 24 h	RT-PCR	Inhibition of cell cycle by DON, 3-AcDON, and 15-AcDON as a result of ATM kinase activation	[43]
		0–10 (µM) for 24, 48, and 72 h	Cell-counting kit-8 (Shanghai, China)	Toxicity ranking below reported: 15-AcDON ≈DON >3-AcDON	
**PBMC from bovine, pig and chicken sources**	DON DOM-1	0–3.37 (µM) for 28 h DON 0–357 (µM) for 72 h DOM-1	Bromodeoxyuridine assay (BrdU)	DOM-1 at a concentration of 357 (µM) inhibits proliferation of PBMC obtained from bovine, pig and chicken sources	[97]

**Table 4:** In vivo assessment of toxicokinetics and toxicity of modified forms of ZEN.

Tested Animals	Tested Toxins	Exposure Type	DOSE	Main Observations	Source
**Pigs**	ZEN-14G ZEN-14S	Intravenously/orally	500 μg/kg BW for ZEN-14G 415 μg/kg BW for ZEN-14S	ZEN-14G may hydrolyse in the circulatory system. ZEN-14G and ZEN-14S are fully hydrolysed within the GI tract	[132]
	ZEN-14G ZEN-14S	Orally	15.1 µg/kg BW for ZEN-14G 12.5 µg/kg BW for ZEN-14S	No detectable quantities of tested toxins were found in urine or faeces	[135]
**Rats**	α-ZOL α-ZOL-14G	Intravenously/orally	0.5 mg/kg BW for α-ZOL 0.75 mg/kg BW for α-ZOL-14G	Efficient conversion of α-ZOL-14G into ZEN. Low bioavailability of oral α-ZOL and α-ZOL-14G after oral administration	[133]
***Caenorhabditis elegans*** **(** **nematodes)**	ZEN ZEN-14S	Medium culture containing mycotoxins	ZEN: 24; 228 (µM) ZEN-14S: 19; 95 (µM)	Comparable reduction in the offspring number of nematodes by ZEN and ZEN-14S	[136]

**Table 5:** In vitro assessment of the toxicity of modified ZEN forms.

Cell Line	Tested Toxins	Applied Concentrations	Methodology	Main Conclusions	Source
**HEPG2**	ZEN α-ZOL β-ZOL	0–100 (µM) for 72 h	Neutral red assay	Established IC50 values(μM): 13.1 for β-ZOL 39.7 forZEN 119 for α-ZOL	[35]
			Qiagen RNeasy midi kit (QIAGEN GmbH, Germany)	IL-1β, IL-8, and TNF-α expression were inhibited by ZEN, α-ZOL, and β-ZOL	
	ZEN α-ZOL	0–250 (μM) for 24 h	MTT	Established IC50 values (µM): 131.40 for α-ZOL 143.35 forZEN	[36]
	ZEN α-ZOL β-ZOL	0–100 (μM) for: 24, 48, and 72 h	MTT	Toxicity ranking: α-ZOL >β-ZOL >ZEN	[124]
	ZEN α-ZOL β-ZOL	0–25 (μM) for 2 h	Fluorescence measured using dichlorofluorescein	Induction of ROS formation by ZEN, α-ZOL, and β-ZOL at all concentrations used	[119]
			Comet assay	Dose-dependent induction of DNA damage by ZEN, α-ZOL, and β-ZOL	
			Spectrophotometry, Ransod (Randox Laboratories, UK)	Increase in the activities of SOD and GPx, decrease in CAT activity upon exposure to ZEN, α-ZOL, and β-ZOL	
	ZEN α-ZOL	0–50 (μM) for 24 h	Western blot	Increased activities of methyltransferase and acetyltransferase. Increased expression of genes coding components of metabolic pathways and nuclear receptors.	[36]
**CACO-2**	ZEN α-ZOL β-ZOL	0–100 (μM) for 48 h	MTT	Established IC50 values (µM): 20 for ZEN 60 forβ-ZOL 80 forα-ZOL	[122]
	ZEN-14G	0–40 (μM) for 6 h	Resazurin dyeing	No ZEN-14G cytotoxicity found	[131]
**SH-SY5Y**	α-ZOL β-ZOL	0–100 (μM) for 72 h	MTT	Established IC50 values (μM): 7.5 for β-ZOL 14 for α-ZOL	[48]
		0–12.5 (μM) for: 24, 48, and 72 h		Synergy in the induction of toxic effect found for the mixture of α-ZOL and β-ZOL	
**Neutrophils isolated from porcine peripheral blood**	ZEN α-ZOL β-ZOL ZAN	0–50 (μM) for 1 h	MTT	Established IC50 values (μM): 53.1 for ZAN 56.8 for β-ZOL 59.0 for α-ZOL 73.4 for ZEN	[138]
		0–10 (μM) for 3 h	ELISA	IL8 expression in neutrophils reduction caused by ZEN, α-ZOL, β-ZOL, and ZAN	
**PBMC isolated from porcine peripheral blood**	ZEN α-ZOL β-ZOL ZAN	0–100 (μM) for 48 h	MTT	Established IC50 values (μM): 17.3 for β-ZOL 22.7 for ZEN 26.3 for ZAN 29.1 for α-ZOL	[139]
		0–10 (μM) for 7 days	ELISA	ZEN, α-ZOL, β-ZOL, and ZAN show the ability to decrease production of antibodies in classes: IgG, IgA, and IgM	
**RAW264.7**	α-ZOL β-ZOL	0–50 (μM) for 24 h	WST-8	Higher cytotoxicity of β-ZOL, compared with that of α-ZOL.	[123]
			Flow cytometry	α-ZOL and β-ZOL induce cell death to a greater extent by apoptosis than by necrosis	
			Flow cytometry Western blot	α-ZOL and β-ZOL induce apoptosis independently of caspases through mitochondrial stress	
**MCF-7**	ZEN-14G	0–1 (μM) for 6 h	MTS	ZEN-14G shows no cytotoxicity	[130]
	ZEN α-ZOL β-ZOL	0–25 (µM) for 6 days	E-Screen	Oestrogen activity displayed by ZEN, α-ZOL, and β-ZOL	[141]
**CHO-K1**	ZEN α-ZOL β-ZOL	0–25 (μM) for 2 h	Fluorescence measured using dichlorofluorescin	Induction of ROS formation by ZEN, α-ZOL, and β-ZOL at all concentrations tested	[149]
			Comet assay	Dose-dependent induction of DNA damage by ZEN, α-ZOL, and β-ZOL	
			Spectrophotometry, Ransod (Randox Laboratories, UK)	SOD and GPx activity increase, CAT activity decreases upon exposure to ZEN, α-ZOL, and β-ZOL	
**Jurkat** **T Cells**	α-ZOL	0–80 (μM) for 24 h	RT–PCR	α-ZOL inhibits expression of IL-2 and IFNγ in a T cell culture	[137]
			3H-thymidine incorporation measurement	T cell proliferation inhibited by α-ZOL	
**HCT116**	α-ZOL β-ZOL	180 (mM) α-ZOL 300 (mM) β-ZOL for 24 h	qRT-PCR	Presence of endoplasmic reticulum stress markers identified upon cell exposure to α-ZOL or β-ZOL	[129]

## Data Availability

Not applicable.
